# In the waiting line: a narrative analysis of patients and caregivers waiting for early intervention psychosis services in Canada

**DOI:** 10.1192/j.eurpsy.2025.331

**Published:** 2025-08-26

**Authors:** F. Poukhovski-Sheremetyev, Y. Pelling, J. Denny, A. Abdel-Baki, S. Iyer, V. Noel

**Affiliations:** 1 Department of Psychiatry, McGill University; 2 Douglas Hospital Research Centre, Montreal; 3 SPOR National Training Entity, Quebec City; 4 Cape Breton University, Cape Breton; 5 Department of Psychiatry and Addictions, University of Montréal, Montreal, Canada

## Abstract

**Introduction:**

Our patients often come to us through wait-lists, but when does the wait really start? Early intervention psychosis programs have introduced policies and benchmarks aimed at minimizing the time between referral and first contact. While programs that meet these targets are often celebrated for their success in reducing untreated psychosis, these targets leave out a critical piece of the puzzle: the time elapsed from symptom onset. Understanding how patients and caregivers experience the wait for care in its entirety is critical to further reducing treatment delays in psychosis.

**Objectives:**

This presentation aims to first examine a disconnect between early intervention wait-list policies and patients’ experiences. Next, it will explore how patient and family member narratives reflect the complex and nuanced nature of waiting for care. Finally, it will propose clinically relevant solutions for reducing the delay between symptom onset and appropriate treatment.

**Methods:**

We conducted individual semi-structured interviews with patients and caregivers accessing early intervention psychosis services across Canada. For this presentation, three interviews from different geographic and socio-cultural regions were selected for their distinct perspectives. We performed a two-reviewer narrative inquiry to derive emergent narratives about waiting for services.

**Results:**

Patient and caregiver experiences revealed two distinct waiting periods. Aside from the “wait-list” period between referral and first contact that is addressed by early intervention policies, participants noted experiencing a much longer initial waiting period, with narratives beginning at symptom onset. Participants described this period as an active, dynamic, frustrating, and often traumatic process that involves multiple ER visits and attempts at receiving care.

**Conclusions:**

We propose formally distinguishing between two forms of waiting for services: passive waiting, which is the state of being on a wait-list, and active waiting, which begins at symptom onset and includes the complex struggle to receive stable care. Early intervention programs’ efforts to reduce passive waiting are important, but the high burden of active waiting suggests a need for larger efforts such as clinician education and systemic changes in how patients access healthcare. Reducing active wait times could truly transform how first episode psychosis is managed and improve outcomes for those in urgent need.

**Disclosure of Interest:**

None Declared

